# Impact of obesity on the outcomes and cost of robotic surgery for Stage IA endometrial cancer: a regional perspective from Japan

**DOI:** 10.1007/s10147-025-02772-8

**Published:** 2025-05-05

**Authors:** Mika Mizuno, Shinichi Togami, Mai Nakazono, Yuriko Higashi, Nozomi Furuzono, Mika Fukuda, Hiroaki Kobayashi

**Affiliations:** Faculty of Medicine, Department of Obstetrics and Gynecology, Kagoshima University, Kagoshima, Japan

**Keywords:** Robotic surgery, Endometrial cancer, Obesity, Surgical outcomes, Medical expenses

## Abstract

**Background:**

The incidence of endometrial cancer in Japan has more than doubled over the past 2 decades because of increasing obesity rates and the unique physiological traits of Asian populations. The aim of this retrospective study was to examine the impact of obesity on surgical outcomes, prognosis, and costs.

**Methods:**

A total of 197 patients with stage IA endometrial cancer who underwent robot-assisted hysterectomy, bilateral salpingo-oophorectomy, and lymphadenectomy/biopsy from 2018 onward were included. Patients were divided into the BMI < 30 kg/m^2^ group (n = 117) and the BMI ≥ 30 kg/m^2^ group (n = 80). The clinical and pathological factors, surgical outcomes, perioperative complications, and treatment costs were compared. The median follow-up period was 34.9 months (range: 6.1–84.2).

**Results:**

In the BMI ≥ 30 kg/m^2^ group, significant differences in comorbidities, including diabetes mellitus (19.7% vs. 51.3%), hypertension (43.6% vs. 58.8%), and hyperlipidemia (29.9% vs. 50%), were detected. However, no significant differences were found in operative time, blood loss volume, perioperative complication rates, or 5-year cancer-specific survival rates (97.6% vs. 100%). Surgical and hospitalization costs were higher in the BMI ≥ 30 kg/m^2^ group, indicating a financial burden for both patients and healthcare facilities. Additionally, a higher prevalence of newly developed lifestyle-related diseases, such as cardiovascular diseases and diabetes, was observed during the follow-up (2.5% vs. 10%).

**Conclusions:**

While obesity (BMI ≥ 30) did not significantly impact surgical outcomes or cancer prognoses, it did increase treatment costs and the risk of lifestyle-related diseases. Thus, preventive strategies, including lifestyle counseling, are needed to reduce obesity-related health burdens.

**Supplementary Information:**

The online version contains supplementary material available at 10.1007/s10147-025-02772-8.

## Introduction

Uterine corpus cancer is one of the most common gynecological malignancies worldwide, with endometrial carcinoma representing most cases. According to GLOBOCAN 2020 data, it is the seventh most common cancer among women, with 417,367 new cases diagnosed and an incidence of 8.7 per 100,000 (age-standardized rate globally) reported in 2020 [1]. Although historically low in Asia, the incidence of endometrial cancer in Japan has increased significantly over the past 20–30 years. In 2000, there were 5,609 new cases, with an age-standardized incidence rate of 8.0 per 100,000 people. By 2020, these numbers had increased to 17,779 cases and an incidence rate of 27.4 per 100,000 people [2]. This trend is linked to lifestyle changes, an aging population, and regional differences in lifestyles and diets. Interestingly, the unique physiological and metabolic characteristics of Asians, particularly their high body fat percentage at a given BMI, is likely a key factor contributing to the increased incidence of EMC among Asians [3–6]. A meta-analysis using CT-based measurements showed that Japanese individuals have significantly greater abdominal visceral fat relative to subcutaneous fat compared to Caucasians, even after adjusting for age and sex (p < 0.05). This suggests ethnic differences in fat distribution that may contribute to metabolic risk [7]. In addition, a large-scale prospective cohort study of approximately 34,000 Japanese women demonstrated that a BMI > 23 is significantly associated with an increased risk of endometrial cancer (HR per 5 kg/m^2^ increase = 1.80; 95% CI, 1.28–2.54). The study also found that weight gain since age 20 and sedentary behavior at the workplace were linked to a higher risk of endometrial cancer [3]. Although the average BMI in Asia is 22.9 kg/m^2^—which is lower than the 26.4 kg/m^2^ reported in Australia and New Zealand—the metabolic risks associated with the distinctive fat distribution in Asian populations remain significant [6]. For example, the risks for cardiovascular disease, hypertension (HT), diabetes mellitus (DM), and EMC are notable even in individuals with BMIs indicating a healthy weight (23–25 kg/m^2^). This has prompted scholars to consider lowering the BMI threshold for obesity in Asian populations. The World Health Organization (WHO) defines obesity as a BMI ≥ 30 (class 1 obesity) [8], whereas in Japan, obesity guidelines classify a BMI > 25 as Grade 1 obesity, with individuals having obesity-related conditions categorized as having “obesity disease” [9].

In terms of treatment, robotic surgery, following laparoscopic surgery, has been widely accepted for EMC in recent years, with many reports emphasizing its effectiveness, particularly in managing patients with obesity [10–17]. In Japan, insurance coverage of laparoscopic and robotic procedures for stage IA EMC has increased, enabling many designated medical institutions to offer these advanced treatment options. Japan’s universal health coverage ensures all residents have access to medical services [18]. Funded primarily through national taxes and premiums paid by citizens, this system provides affordable medical care, with patients responsible for copayments ranging from 0 to 30% of the total cost. Additionally, out-of-pocket expenses are capped, allowing equitable access to expensive treatments, including advanced surgeries and high-cost drugs. Hospitalization costs are determined by two systems: Diagnosis Procedure Combination (DPC) and fee-for-service (FFS). With the DPC system, a fixed daily rate is set on the basis of the patient’s diagnosis and treatment, covering most inpatient services, whereas with the FFS system, each procedure or treatment is charged individually. With this system, costs are generally standardized if the same surgical procedure is performed for the same disease under the fixed-point system.

At our institution, which serves local cities and remote islands, the median BMI of stage IA EMC patients treated in the past five years was 28, indicating a higher obesity rate than in other facilities in Japan. In this study, we retrospectively analyzed the impact of obesity on the outcomes and cost of treatment for stage IA EMC to identify challenges and propose solutions for perioperative management in the context of obesity.

## Materials and methods

Between April 2018 and September 2023, 301 patients diagnosed preoperatively with stage IA grade 1–2 EMC underwent surgery at our institution. The median BMI was 27.3 (range: 16.4–53.1). Among these patients, 24 (7.9%) underwent open surgery, 67 (22.3%) underwent laparoscopic surgery, and 210 (69.7%) underwent robotic surgery. A total of 197 robotic-assisted hysterectomies (RHs) performed using the da Vinci® surgical system were analyzed in this study. Patients who were converted to open surgery due to severe adhesions (n = 6) and those who underwent robotic surgery with another robotic system (hinotori™, n = 7) were excluded from the analysis.

This study was approved by the Ethics Committee of Kagoshima University (approval number: 220184) prior to registration. Informed consent was obtained through an opt-out system, ensuring the protection of participants’ rights and autonomy. The study was conducted in adherence to the principles outlined in the Declaration of Helsinki.

Patients were classified into two groups on the basis of their BMI: < 30 kg/m^2^ and ≥ 30 kg/m^2^. The clinical characteristics, surgical outcomes (operative time, perioperative complications, and prognosis), and hospitalization costs, including surgical fees, were assessed and compared between the two groups. Perioperative complications were evaluated using the Common Terminology Criteria for Adverse Events (CTCAE) ver.5. translated by the Japan Clinical Oncology Group (JCOG), and postoperative complications were classified using Clavien-Dindo classification ver.2, developed by JCOG.

Statistical analyses were performed using IBM® SPSS® Statistics ver.24. The chi-square test, t test, and Mann–Whitney U test were used for comparisons between the two groups. Survival rates were evaluated via Kaplan‒Meier survival curves.

## Results

### Patient characteristics

**Fig. 1:**
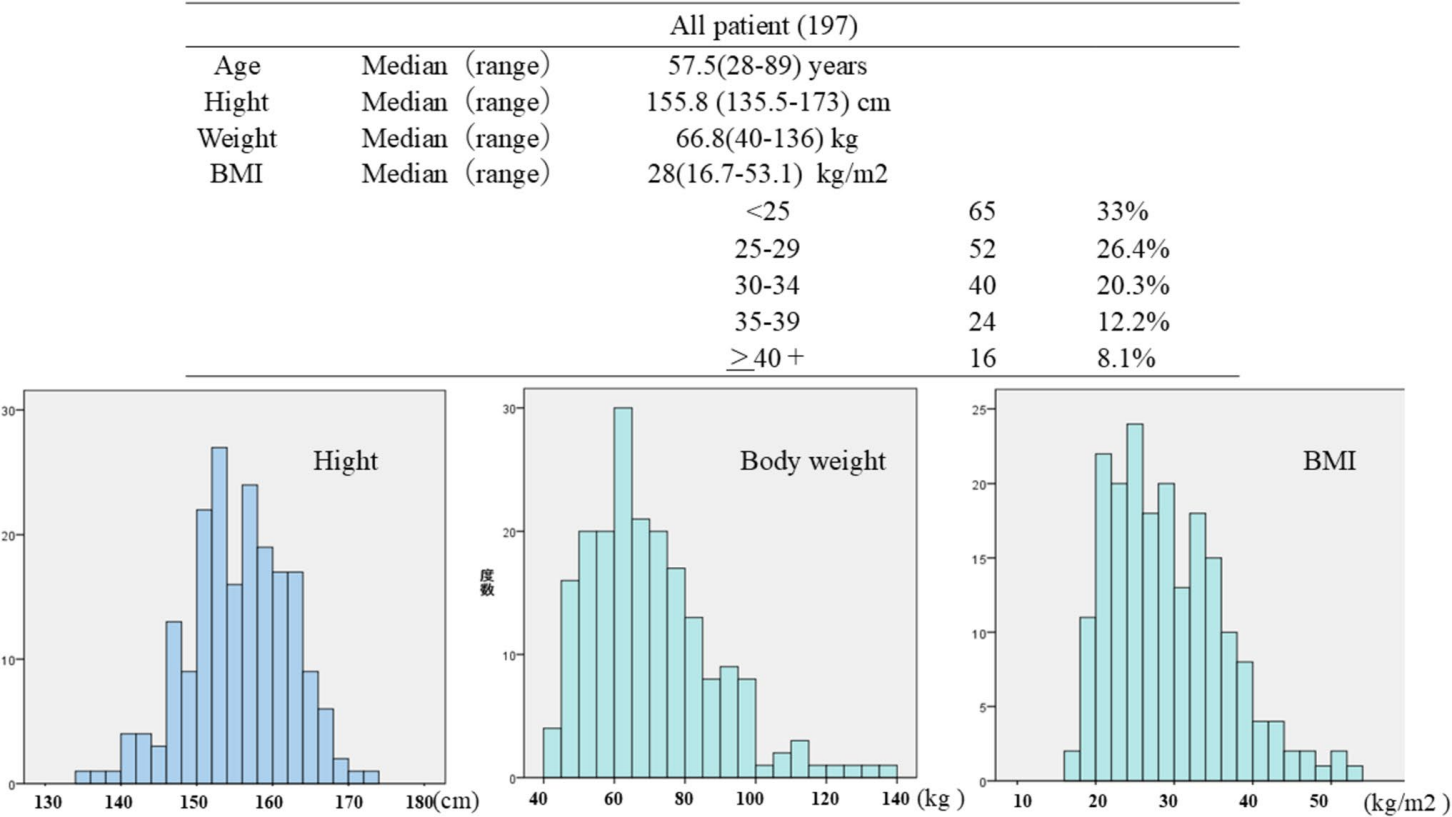
Body composition distribution of all cases

The median values for age, height, weight, and BMI for the entire cohort of 197 patients were 57.5 years (range: 28–89), 155.8 cm (range: 135.5–173), 66.8 kg (range: 40–136), and 28 kg/m^2^ (range: 16.7–53.1), respectively. Patients were classified into categories on the basis of their obesity status determined by the BMI. Among the patients, 67% had a BMI > 25, and 20% had a BMI > 35. Moreover, 18.2% had a BMI < 23, and 14.7% had a BMI between 23 and 24. The distributions of body weight and BMI are illustrated in Fig. 1.

The demographics and clinical characteristics of the patients in the two groups, the BMI < 30 (n = 117) group and the BMI ≥ 30 (n = 80) group, are summarized in Table 1. Significant differences were observed in age, parity, sexual experience, and preoperative pathological diagnosis between the two groups. With respect to comorbidities, DM, HT, and hyperlipidemia (HL) were significantly more prevalent in the BMI ≥ 30 group, with DM being particularly notable (51.3%). The American Society of Anesthesiologists Physical Status (ASA-PS) classification indicated a significantly higher predicted risk in the BMI ≥ 30 group, reflecting the increased prevalence of obesity and associated comorbidities. Both groups had a similar history of prior abdominal surgeries, with approximately 40% of patients in each group, and no significant differences were observed in this parameter.

**Table 1:** Patient demographics and clinical characteristics

Variables	BMI < 30 (n = 117)		BMI ≥ 30 (n = 80)		*P*
	N	%	N	%	
Median Age (y.o.)	58 (32–89)	56 (28–86)	0.019		
Median Height (cm)	156 (137–173)	153.5 (135.5–167.1)	0.190		
Parity	82	70.1	40	50	0.003
Sexual experience	107	91.5	60	75	0.0017
Preoperative diagnosis					
Atypical endometrial hyperplasia	0	0	6	7.5	0.004
Endometrial cancer stage IA	117	100	74	92.5	
Comorbidities					
Diabetes mellitus	23	19.7	41	51.3	< 0.0001
Hypertension	51	43.6	47	58.8	0.026
Hyperlipidemia	35	29.9	40	50	0.003
Psychiatric disorder	13	11.1	8	10.2	0.488
Antithrombotic agents	9	7.7	5	6.4	0.484
Past medical history					
Abdominal surgery	51	43.6	27	33.8	0.108
Malignant disease	18	15.4	10	12.5	0.362
Lifestyle history					
Smoking history	15	12.8	12	15	0.407
Alcohol consumption	16	13.8	3	3.8	0.016
ASA-PS classification*					
1	50	42.7	0	0.0	< 0.001
2	56	47.9	50	64.1	
3	10	8.5	28	35.9	
4	1	0.9	0	0.0	

All items: Yes *American Society of Anesthesiologists Physical Status

### Surgical outcomes

A comparison of factors associated with surgery is presented in Table 2.

**Table 2:** Comparison of Factors Associated with Surgery

Variables	BMI < 30 (n = 117)		BMI ≥ 30 (n = 80)		*P*
	N	%	N	%	
Primary surgical procedure					
RH + BSO + PLN biopsy (SNNS)	100	85.5	64	80.0	0.08
RH + BSO + PLN dissection (± SNNS)	14	12.0	8	10.0	
RH + BSO	3	2.6	8	10.0	
Additional surgeries					
Lower abdominal mini-laparotomy^a^	5	4.3	2	2.5	0.282
Episiotomy and vaginal wall laceration with suturing^a^	10	8.5	6	7.5	
Vaginal wall reconstruction for pelvic organ prolapses	3	2.6	0	0.0	
Others^b^	9	7.7	5	4.9	
Surgical time Median [range]					
Total operative time, min	200 (101–348)	209 (84–555)	0.402		
Console time, min	151 (70–267)	155 (53–453)	0.483		
Open/closure time, min	46 (11–150)	44 (8–104)			
Blood loss volume Median (range)g	20 (5–335)	20 (2–453)	0.315		
Postoperative length of hospital stayMedian (range) days	5 (4–35)	5 (4–15)	0.947		

*RH* Robot-assisted hysterectomy, *BSO* Bilateral salpingo-oophorectomy, *PLN* Pelvic lymph node, *SNNS* Sentinel lymph node navigation surgery, *PLN biopsy (SNNS)* sentinel node biopsy only, *PLN dissection (± SNNS)* lymphadenectomy performed due to undetected sentinel nodes or positive sentinel nodes metastasis. ^a^Procedures related to the retrieval of the resected uterus, ^b^Repair of organ injury, severe adhesion detachment, ovarian or vulvar tumor resection

Approximately 80% of patients in both groups underwent RH, bilateral salpingo-oophorectomy (BSO), and pelvic lymph node biopsy with sentinel lymph node navigation surgery, whereas approximately 10% underwent pelvic lymphadenectomy. No significant differences were observed between the groups regarding additional intraoperative procedures, such as mini-laparotomy for uterus retrieval, perineal or vaginal wall incisions, or vaginal wall lacerations. Similarly, there were no significant differences in operative time, blood loss volume, or postoperative length of hospital stay between the groups.

### Perioperative complications

A summary of perioperative complications requiring medical intervention is presented in Table 3 according to BMI group (< 30 vs. ≥ 30). Intraoperatively, 1.5% of patients experienced CTCAE grade 1 events, and 0.5% experienced grade 2 events. Postoperatively, 0.5% experienced grade I, 9.1% grade II, and 0.5% grade IIIa complications. None of the patients required adjustment of the Trendelenburg position (25–30 degrees) due to pulmonary ventilation issues. Three patients were admitted to the ICU as a precaution due to severe obesity or comorbidities, but all recovered without severe complications. There was no significant difference in the overall incidence of intraoperative or postoperative adverse events between the BMI groups (p = 0.699 and p = 0.262, respectively). However, patients with a BMI > 35 had a significantly higher rate of postoperative infectious complications (17.5% vs. 7%), with an odds ratio of 2.82 (95% CI: 1.02–7.80, p = 0.047).

**Table 3:** Perioperative Complications

	BMI < 30 (n = 117)		BMI ≥ 30 (n = 80)		P
	N		N		
**Intraoperative**	**CTCAE** ^a^				
Rectal injury		–	G2	1 (1.3%)	0.699
Small bowel injury	G1	1 (0.9%)		–	
Bladder injury	G1	1 (0.9%)		–	
Bleeding at port insertion site		–	G1	1 (1.3%)	
**Postoperative**	**Clavien‒Dindo Classification**^b^				
Ureteral injury/stenosis	III^a^	1 (0.9%)		–	0.262
Pelvic infection ^c^	II	6 (5.1%)	II	7 (8.7%)	
Urinary tract infection	II	1 (0.9%)	II	1 (1.3%)	
Lower extremity cellulitis		–	II	1 (1.3%)	
Vaginal stump bleeding/separation		–	II	1 (1.3%)	
		–	I	1 (1.3%)	
Delirium	II	1 (0.9%)		–	

^a^*CTCAE* Common Terminology Criteria for Adverse Events. ver.5, ^b^*Clavien-Dindo Classification* A system for grading surgical ver.2, ^c^Including 1 case of lymphocyst infection

### Post-treatment outcomes

Postoperative staging and adjuvant therapy are presented in Supplementary Table 1, with no significant differences observed between the groups. Among all the cases, 82.7% were classified as stage IA (including two cases of AEH that were upstaged to EMC), 7.1% as stage IB, 2.0% as stage II, 2.5% as stage IIIA, and 3.5% as stage IIIC. Adjuvant therapy was administered to 17.8% of patients, including chemotherapy to 28 patients and radiation therapy to 7 patients.

The median follow-up period was 34.9 months (range: 6.1–84.2). The posttreatment outcomes are summarized in Table 4. EMC recurred in 5 patients, 2 of whom died. The remaining 3 patients exhibited remission after treatment. Among the five patients who experienced recurrence, four had identifiable pathological risk factors, including stage IIIA (peritoneal dissemination in the pouch of Douglas), stage IIIC (lymph node metastasis), stage II (cervical stromal invasion), and stage IA with positive ascites cytology. There was no significant difference in the 5-year disease-free survival rate between the BMI < 30 and BMI ≥ 30 groups (97.1% vs. 96.7%, p = 0.986), and the 5-year cancer-specific survival rate was also comparable between the two groups (97.6% vs. 100%, p = 0.255; Supplementary Fig. 1). Six patients (3%) developed new malignant diseases during the follow-up period, with no significant difference between the groups. However, the BMI ≥ 30 group showed a trend toward a higher incidence of severe lifestyle-related diseases requiring treatment with or without hospitalization than the BMI < 30 group (8 vs. 3 patients; 10% vs. 1.5%, p = 0.055). Both patients who required dialysis had BMIs of 23.7 and 27.7, respectively, and suffered from renal failure caused by DM. Additionally, thrombotic events included severe pulmonary thromboembolism and cerebral infarction.

**Table 4:** Outcomes of All Patients

Clinically Significant Events Requiring Treatment	N, %		BMI < 30 (n = 117)	BMI ≥ 30 (n = 80)	*P*
EMCa recurrence (including pT2,3)	5	2.5%	3	2	1.0
Cancer-specfic death	2	1%	2	0	
Disease-free survival	3	1.5%	1	2	
Other organ cancer	6	3%	5	1*	0.429
Lifestyle-related diseases	11	5.6%	3	8	0.055
Initiation of dialysis	2	1%	2	0	
Thrombosis	2	1%	0	2	
Diabetes, liver disease, etc	7	3.5%	1	6	
Fractures/orthopedic surgery	3	1.5%	1	2	-
Others	2	1%	2	0	-
Total	27		13	14	

*EMCa* endometrial cancer, *A* A patient with a BMI of 53 developed lung cancer. Subsequently, their BMI increased to 60, leading to worsening respiratory impairment due to severe obesity. This made treatment challenging, and the patient unfortunately passed away

One notable case involved a 61-year-old patient with mild intellectual disability and a BMI of 53 who experienced severe respiratory issues and had poor weight control, leading to the cancellation of planned surgery. After weight loss, the patient underwent RH with good outcomes but later regained weight, reaching a BMI of 60. The patient developed early-stage lung cancer 2 years later, but severe obesity and respiratory impairment limited treatment, ultimately resulting in death.

### Medical cost

As mentioned, the total cost of EMC surgery consists of the hospitalization fee under the DPC system and the surgery fee, including anesthesia, drugs, and procedures required during surgery, under the FFS system. Moreover, medications administered and procedures performed in the ward before or after surgery are included in the fixed DPC fee. This system ensures both efficiency and transparency in terms of healthcare costs.

Table 5 presents the hospitalization costs (in Japanese yen) for EMC patients who underwent RH over a two-year period, categorized by BMI < 30 and BMI ≥ 30. The costs billed under the DPC system (actual billed amounts) were compared with those re-calculated solely under the FFS system (reference values). The average costs were significantly higher in the BMI ≥ 30 group, under both the DPC and FFS system (p < 0.05). The average difference between DPC and FFS costs was -¥6,692 in the BMI < 30 group and -¥18,139 in the BMI ≥ 30 group—approximately 2.7 times greater. Although not statistically significant, the cost difference was larger in the BMI ≥ 30 group. The difference between these two cost systems represents an indirect financial burden that the hospital must bear. Additionally, the average additional surgical cost, which reflects actual charges, was ¥24,074 in the BMI < 30 group and ¥35,172 in the BMI ≥ 30 group, revealing a 1.46-fold higher cost in the latter.

**Table 5:** Comparison of Total Hospitalization Costs: The DPC System (Actual Billed Amounts) vs. Recalculated Using FFS (Reference Values)

Variables	(1) BMI < 30 (n = 60)	(2) BMI ≥ 30 (n = 42)	(2)—(1)	*P*
A: DPC (JPY) Mean ± SE	A1	A2	+ 34,142.9 ± 1,663	0.043
95%CI			116–6,713	
B: FFC (JPY)^a^				
Mean	B1	B2	+ 36,275.3 ± 1,767	0.040
95%CI			121–7,134	
B-A (JPY)				
Mean	– 6692.5 ± 19,861.6	– 18,139.5 ± 4242.5	– 11,450 ± 7640	0.137
95%CI			– 2,661—372	

Total hospitalization costs in Japanese yen are determined using the Diagnosis Procedure Combination (DPC) and fee-for-service (FFS), a represents an estimated amount recalculated entirely under the FFS system. The actual values of A1, A2, B1, and B2 are confidential information; therefore, only the differences between the two groups are presented in the table. *SE* standard error, *95%CI* 95% Confidence Interval, a represents an estimated amount recalculated entirely under the FFS system

### Preoperative approaches for obese patients

In three patients, surgeries were postponed due to poor weight and diabetes control, prompting a focus on optimizing preoperative conditions. In addition to routine exams, comprehensive assessments were introduced for patients with HbA1c > 7.5 or newly diagnosed DM, such as intensified insulin therapy. During the 2–3 month waiting period before surgery, with strategies such as family-accompanied nutritional counseling, exercise guidance, follow-up calls implemented for those with mild-to-moderate obesity, and hospitalization for those with severe obesity. Anesthesiologists have also evaluated perioperative risks. As a result of these measures, among 48 patients given weight loss instructions (median weight: 93.7 kg; range: 69–139 kg), 37 actively participated, achieving a median weight loss of 4.25 kg (range: 1–22 kg), with 5 losing 10 kg or more, enabling surgeries to be performed without complications.

## Discussion

Obesity is a well-established risk factor for EMC, and many studies have revealed that the average BMI of patients undergoing surgery for EMC exceeds 30 [10, 13–17, 19–26]. According to 2017–2018 data from the National Health and Nutrition Examination Survey, 27.5% of American women are overweight (BMI 25–29), and 41.9% are obese (BMI > 30) [27]. In contrast, the average BMI of Japanese women is approximately 22, and 22.3% have a BMI > 25, with no significant change over the past two decades.

Reports from Japan show that the average BMI of EMC patients ranges from 23 to 26 [28, 29], similar to other East Asian populations [30, 31].

In EMC patients, HT is reported in more than 50% of those with a BMI > 30 and 58–74% of those with a BMI > 40, although significant differences according to obesity category are often not observed [13, 20, 26, 32, 33]. In contrast, the prevalence of DM varies across studies depending on BMI, ranging from 21.6% to 39.5% for individuals with a BMI > 30 [13, 20, 26, 32, 33]. In this study, the prevalence of DM was 51.3% in individuals with a BMI > 30, similar to Japanese data revealing a prevalence of 46% in individuals with a BMI 30–39 [21]. While the prevalence of DM is typically reported to be 7–18% in individuals with a BMI < 30 [21, 26, 34, 35], our data revealed a higher prevalence of 35.8%, even in patients with a BMI ranging from 25–29. These findings suggest that Japanese patients tend to have a higher prevalence of DM than other populations do, especially if they have a BMI > 25.

Obesity is generally associated with worse surgical outcomes, and the incidence of complications tends to increase with increasing BMI [36]. However, many reports have revealed that RA, as a minimally invasive surgery, is associated with a lower incidence of complications than conventional laparotomy is and can be performed safely and effectively in obese patients [5, 12, 16, 17, 23, 24, 36, 37], but the operation time is longer than that of conventional laparotomy [12, 23, 38]. In our hospital, the operative time tended to be shorter for minimally invasive procedures than for open surgery, possibly due to a high level of surgical experience and reduced closure times in obese patients (data not shown). Many studies have revealed that there are no significant differences in operative outcomes or perioperative complication rates of RA, regardless of BMI [11, 13, 19, 20, 26, 33]. Our data similarly revealed differences between obese patients (BMI > 30) and those with a BMI < 30. Moreover, some have noted that the incidence of complications increase with increasing BMI [37], as evidenced by a higher incidence of wound infections in patients with a BMI > 40 [13]. Similarly, our data revealed a significantly higher rate of Class II postoperative pelvic infections in patients with a BMI > 35.

Survival outcomes for EMC are generally favorable, with many reports indicating no significant differences between laparotomy and laparoscopic surgery [14, 15, 25]. In addition, a prospective study conducted in Australia investigated the long-term prognosis of 760 obese cancer patients who underwent laparotomy or laparoscopic surgery, with approximately 60% having a BMI of 30 or more and a follow-up period of 4.5 years [24]. The study revealed no difference in recurrence rates, which were approximately 8%. Across all the cases, new cancers were observed in approximately 8.4% of patients, with 3.9% of deaths attributed to EMC and 3.1% attributed to other causes. Similarly, our data revealed a relatively favorable survival outcome for EMC, with a mortality rate of 1% during a median follow-up period of 3 years. However, research suggested that new cancers, as well as obesity- and diabetes-related conditions such as cardiovascular disease and renal failure, may impact long-term survival outcomes.

In terms of costs, while robotic surgery is highly effective, many reports highlight its higher cost compared to laparotomy and laparoscopic surgery, raising concerns about the financial burden on healthcare systems [12, 17, 19, 23, 39, 40]. On the other hand, studies have revealed that higher annual surgical volumes could improve cost-effectiveness [12], and robotic surgery in patients aged 70 and older has been reported to provide overall good cost-performance [35]. Our healthcare cost data revealed that both the actual billed amount and surgical costs were significantly higher for patients with a BMI ≥ 30. As previously mentioned, the difference between the costs billed under the DPC system (actual billed amounts) and those recalculated under the FFS system (reference values) was greater in patients with a BMI ≥ 30 than in those with a BMI < 30. This is likely due to an increase in non-billable medical procedures under the DPC system, such as the additional use of medications, intravenous fluids, and interventions required for higher body weight and comorbidities. These findings suggest that patients with a BMI ≥ 30 may impose a greater financial burden not only on themselves but also on hospitals and national healthcare resources. Furthermore, we speculate that non-quantifiable factors—such as the increased workload for healthcare staff related to patient transfer and repositioning—may also contribute to the overall cost.

In this study, robotic surgery was performed safely across BMI categories, suggesting that obesity alone may not adversely impact surgical safety in a well-managed perioperative setting. This raises the question: Is preoperative weight loss truly necessary? While our data did not show a direct link between weight reduction and surgical outcomes, previous studies have demonstrated that morbid obesity (BMI > 40) is associated with an increased risk of perioperative complications. Additionally, anesthesiologists and gynecologic surgeons may experience greater psychological and physical stress when managing severely obese patients in the steep Trendelenburg position. As noted earlier, preoperative weight loss may help reduce the use of medical resources, prevent the progression of obesity-related comorbidities, and lessen the physical burden on healthcare personnel. Therefore, we believe that preoperative weight management remains an important element of comprehensive perioperative care in patients with obesity.

At the same time, while this study does not propose specific policy recommendations, our findings suggest that investing in public health efforts to prevent obesity and lifestyle-related diseases may ultimately be more cost-effective than focusing solely on the treatment of complications using advanced medical technologies. In light of these findings, we believe that future healthcare policies should consider balancing investments in high-cost treatments with broader public health initiatives aimed at preventing obesity and improving population health.

The limitations of this study include its retrospective design, use of single-institution data, and its relatively small sample of cases. Healthcare costs cannot be definitively assessed only on the basis of these data, and larger studies are needed. However, with an average height of 155.3 cm and a high proportion of obese patients, our findings offer valuable insights into the outcomes and cost of robotic surgery for obese EMC patients, particularly in Japanese and Asian populations.

## Conclusion

In this study, patients with a BMI > 30 tended to have higher rates of comorbidities, such as DM, HT, and HL, but there were no significant differences in surgical outcomes or perioperative complications. These findings suggest that robotic surgery and comprehensive preoperative management may be effective in mitigating perioperative risks. However, healthcare costs were higher for obese patients, who also appeared to have a higher incidence of severe lifestyle-related diseases after treatment, indicating the importance of preventive measures targeting obesity and related conditions.

## Supplementary Information

Below is the link to the electronic supplementary material.

Supplementary file1 (TIF 98 KB) Supplementary Fig. 1; Kaplan–Meier survival curves for patients stratified by BMI group (<30 vs. ≥30), A: 5-year disease-free survival rate, B: 5-year cancer-specific survival rate

Supplementary file2 (DOCX 18 KB)

## Data Availability

All the data generated or analyzed during this study are included in this article. Further inquiries can be directed to the corresponding author.
