# Supplementary figures and images for: Impact of obesity on the outcomes and cost of robotic surgery for Stage IA endometrial cancer: a regional perspective from Japan
Source: Int J Clin Oncol. 2025 May 5;30(7):1426–35. doi: 10.1007/s10147-025-02772-8 (PMC12187788; doi:10.1007/s10147-025-02772-8)

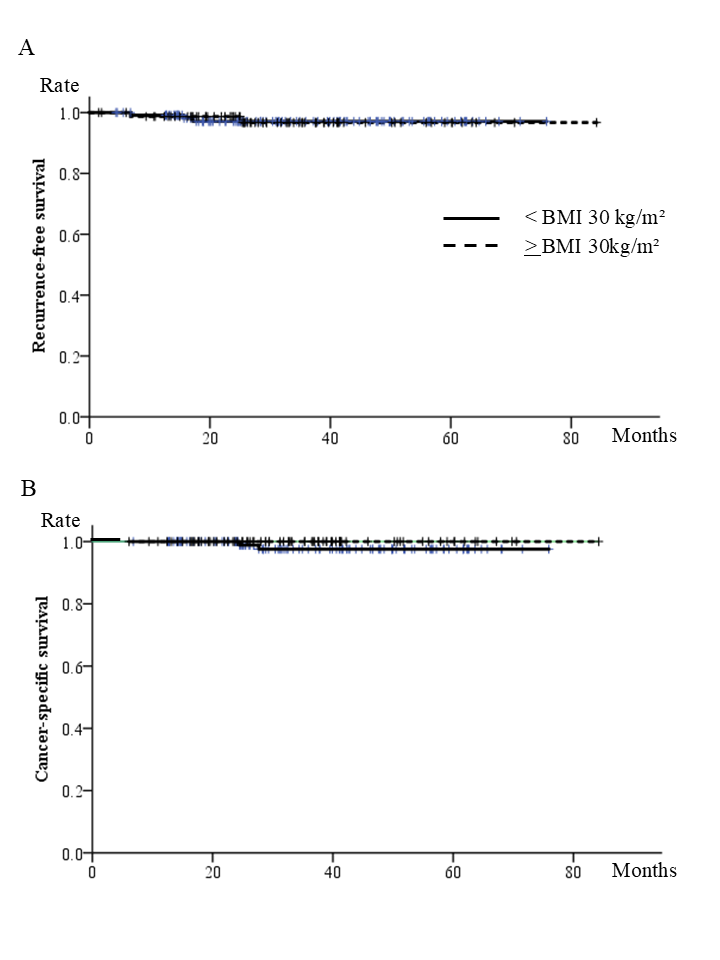

Supplement: Supplementary file 1 — Supplementary file1 (TIF 98 KB) Supplementary Fig. 1; Kaplan–Meier survival curves for patients stratified by BMI group (<30 vs. ≥30), A: 5-year disease-free survival rate, B: 5-year cancer-specific survival rate [file 10147_2025_2772_MOESM1_ESM.tif]
