# Supplementary material for: Impact of obesity on the outcomes and cost of robotic surgery for Stage IA endometrial cancer: a regional perspective from Japan
Source: Int J Clin Oncol. 2025 May 5;30(7):1426–35. doi: 10.1007/s10147-025-02772-8 (PMC12187788; doi:10.1007/s10147-025-02772-8)
Supplement: Supplementary file 2 — Supplementary file2 (DOCX 18 KB) [file 10147_2025_2772_MOESM2_ESM.docx]

**Supplementary Table 1.** Comparison of postoperative factors and treatment

| **Postoperative factors** | | BMI＜30 (n=117) | | BMI>30 (n=80) | |  |
| --- | --- | --- | --- | --- | --- | --- |
|  |  | n | % | n | % | P |
| **TNM classification** | |  | |  | |  |
| **AEH** |  | 0 | 0.0 | 4 | 5 | **0.213** |
| **pT** | 1a | 100 | 85.5 | 63 | 78.8 |  |
|  | 1b | 4 | 3.4 | 10 | 12.5 |  |
|  | 2 | 4 | 3.4 | 0 | 0 |  |
|  | 3 | 9 | 7.7 | 3 | 3.8 |  |
| **N** | 0 | 108 | 92.3 | 71 | 88.8 | 0.121 |
|  | 1 | 6 | 5.1 | 1 | 1.3 |  |
|  | X | 3 | 2.6 | 8 | 10 |  |
| **Adjuvant therapy** | |  |  |  |  |  |
| Yes |  | 20 | 17.1 | 15 | 18.8 | 0.767 |
|  | Chemotherapy | 16 |  | 12 |  |  |
|  | Radiation | 4 |  | 3 |  |  |
| TNM Classification, 5th Edition (2020), AEH: atypical endometrial hyperplasia | | | | | | |
